# The impact of lockdown in Wuhan on residents confidence in controlling COVID-19 outbreak at the destination cities

**DOI:** 10.3389/fpubh.2022.902455

**Published:** 2022-08-15

**Authors:** Xiaoxin Guo, Shihu Zhong, Yidong Wu, Yalin Zhang, Zhen Wang

**Affiliations:** ^1^Institute of Applied Economics, Shanghai Academy of Social Sciences, Shanghai, China; ^2^Shanghai National Accounting Institute, Shanghai, China; ^3^School of Business, Anhui University of Technology, Ma'anshan, China; ^4^School of Economics, Nanjing University of Posts and Telecommunications, Nanjing, China; ^5^School of International and Public Affairs, Shanghai Jiao Tong University, Shanghai, China

**Keywords:** population inflow, controlling COVID-19, destination cities, China, confidence

## Abstract

**Objective:**

From January 23rd, 2020, lock-down measures were adopted in Wuhan, China to stop the spread of COVID-19. However, due to the approach of the Spring Festival and the nature of COVID-19, more than 6 million permanent and temporary residents of Wuhan (who were potential carriers or spreaders of the virus), left the city before the lock-down measures were implemented. This study aims to explore whether and how the population inflow from Wuhan city impacted residents' confidence in controlling COVID-19 outbreaks at the destination cities.

**Study design and setting:**

Based on questionnaire data and migration big data, a multiple regression model was developed to quantify the impact of the population inflow from Wuhan city on the sense of confidence of residents in controlling the COVID-19 outbreak at the destination cities. Scenarios were considered that varied residents' expected month for controlling COVID-19 outbreak at the destination cities, residents' confidence in controlling COVID-19 outbreak at the destination cities, and the overall indicators for the sense of confidence of residents in controlling COVID-19. A marginal effect analysis was also conducted to calculate the probability of change in residents' confidence in controlling the COVID-19 outbreak with per unit change in the population inflow from Wuhan city.

**Results:**

The impact of population inflow from Wuhan city on residents' expected month for controlling COVID-19 outbreak at the destination cities was positive and significant at the 1% level, while that on residents' confidence in controlling COVID-19 at the destination cities was negative and significant at the 1% level. Robustness checks, which included modifying the sample range and replacing measurement indicators of the population inflow from Wuhan city, demonstrated these findings were robust and credible. When the population inflow from Wuhan city increased by one additional unit, the probabilities of the variables “February” and “March” decreased significantly by 0.1023 and 0.1602, respectively, while the probabilities of “April,” “May,” “June,” “July,” “before the end of 2020,” and “unknown” significantly increased by 0.0470, 0.0856, 0.0333, 0.0080, 0.0046, and 0.0840, respectively. Similarly, when the population inflow from Wuhan city increased by one additional unit, the probability of the variable “extremely confident” decreased by 0.1973. Furthermore, the probabilities of the variables “confident,” “neutral,” and “unconfident” significantly increased by 0.1392, 0.0224, and 0.0320, respectively.

**Conclusion:**

The population inflow from Wuhan city played a negative role in the sense of confidence of residents in controlling COVID-19 in the destination cities. The higher the population inflow from Wuhan city, the longer the residents' expected month for controlling COVID-19 outbreak at the destination cities became, and the weaker the residents' confidence in controlling the COVID-19 outbreak at the destination cities.

## Introduction

The world is still suffering from a global pandemic of novel coronavirus (COVID-19). This has become a significant public health threat to the wellbeing and social stability of people on a global scale (1–13). As of May 29, 2022, 2,748 cases of COVID-19 have been confirmed in China with 5,226 deaths recorded. Outside of China, there have been roughly 531,101,352 confirmed cases of the disease and more than 6,310,100 deaths have been reported. Corona Virus Disease 2019 (COVID-19), referred to as “novel coronavirus pneumonia” and named “coronavirus disease 2019” by the World Health Organization, refers to pneumonia caused by COVID 19 (https://www.who.int/emergencies/diseases/novel-coronavirus-2019). According to the available case data, novel coronavirus pneumonia is mainly manifested by fever, dry cough, and malaise, and a few patients have upper respiratory and gastrointestinal symptoms such as nasal congestion, runny nose, and diarrhea. Since the emergence of COVID-19 in China, the country has adopted strict prevention and control measures in a bid to curb the outbreak of the disease (14–16). On January 23rd, 2020, Wuhan adopted lock-down measures. The operation of buses, metros, ferries, and long-distance coaches ceased. Public transportation facilities, such as airports and railway stations for people leaving Wuhan were also shut down. However, due to the approach of the Spring Festival and the nature of COVID-19, more than 6 million permanent and temporary residents of Wuhan (who were potential carriers or spreaders of the virus), left the city before the lock-down measures were implemented (January 10th−24th, 2020). On January 25, 2020, out-migration population in Wuhan began to converge to zero. As severe acute respiratory syndrome coronavirus 2 (SARS-CoV-2) carriers traveled to countries or regions free of sustained transmission, they may have affected the transmission of COVID-19 in those countries and regions (17, 18). Existing research reveals a correlation between population outflow from Wuhan and the number of people diagnosed with COVID-19 (5, 14, 15, 19). And some studies have revealed that the Wuhan lockdown could benefit many people and communities, including the locals and the others (12, 20–23), and substantially suspends the national and global outbreak of COVID-19 pandemic (7, 24–26). Moreover, dynamical modeling is one of the useful tools to reveal the transmission dynamics of COVID-19 (27–30). Sun et al. (31) employed the dynamical model to investigate the effects of lockdown on the COVID-19 transmission in Wuhan, and found that although a later adoption of lockdown measures would reduce the scale of the epidemic in this city, there would be uncontrollable effects on other Chinese provinces and even the world. Besides, some researchers systematically explore the economic, social, and mental health impacts of COVID-19 (32–34). For example, Gautam et al. (32) investigated the impact of COVID-19 on mental health and found that women face more depression and anxiety than men, as well as 43% of children, had subthreshold mental disturbances.

As the outbreak of COVID-19 occurred throughout China and across the globe, fear of the pandemic is also spreading. The confidence of people is a sign of early victory over the disease and directly affects their morale, which in turn causes disease prevention and stability of the overall society. Therefore, temporary closure of Wuhan city effectively slowed the spread of the COVID-19 at the time, which may have affected people's confidence in the early production of the virus. But, few studies have examined whether and how lockdown in Wuhan city affects residents' confidence in controlling the COVID-19 outbreak. Hence, from the perspective of the sense of confidence of residents in controlling COVID-19 in the destination cities, this study attempts to provide evidence for the significance of the temporary closure of Wuhan city.

Based on questionnaire data and migration big data, we employ a multiple regression model to quantify the impact of the population inflow from Wuhan city on residents' expected month for controlling COVID-19 outbreak at the destination cities, residents' confidence in controlling COVID-19 outbreak at the destination cities, and the overall indicators for the sense of confidence of residents in controlling COVID-19. Moreover, we also use a marginal effect analysis to calculate the probability of change in residents' confidence in controlling the COVID-19 outbreak with per unit change in the population inflow from Wuhan city.

Specifically, a questionnaire, titled “Questionnaire on community and pandemic perception under COVID-19,” was designed to investigate the subjective feelings and expectations of residents under the influence of COVID-19. Using the questionnaire, a nationwide online survey was conducted between February 10th and February 25th, 2020 to collect relevant data. We clarify that the Questionnaire was conducted for this study purpose. The data collected covered 31 provinces, municipalities, and autonomous regions in mainland China, as well as the Hong Kong Special Administrative Region, and Taiwan. A total of 1,060 questionnaires were distributed, of which 1,049 valid questionnaires were obtained and 9.06% of those were collected from the hardest-hit Hubei Province. An estimate of the population inflow from Wuhan to the rest of the country before the Spring Festival (January 10th−24th, 2020) was also made. Finally, an investigation was conducted on the relationship between the population inflow from Wuhan city and the sense of confidence of residents in controlling the COVID-19 outbreak in the destination cities.

Our study contributes to the existing literature in the following aspects. On the one hand, this study provides the empirical identification of the impact of lockdown in Wuhan on residents' confidence in controlling the COVID-19 outbreak in the destination cities. Previous literature mainly explores the effects of lockdown on the spread of COVID-19, while few research studies explored its impact on the psychological aspects of people. On the other hand, by exploiting the questionnaires and migration big data in China, we discover that the higher the population inflow from Wuhan city, the longer the residents' expected month for controlling COVID-19 outbreak at the destination cities became, and the weaker the residents' confidence in controlling COVID-19 outbreak at the destination cities. Such findings help to enrich the literature on both the COVID-19 outbreaks specifically and outbreaks in general.

## Methods

### Model structure

To investigate the impact of the population inflow from Wuhan city on the sense of confidence of residents in controlling the COVID-19 outbreak at the destination cities, the panel data regression model was constructed as equation (1). Panel data regression models refer to regression models that include both time dimension and cross-sectional dimension data. The advantage is that it is possible to take into account both the commonalities that exist in cross-sectional data and to analyze the individual specific effects of cross-sectional factors in the model. However, panel data regression models require high data quality.


(1) $$\begin{array}{l} SOC_{ijt}=\beta_{0,1}+\beta_{1,1}Wuhan\_inflow_{ijt}\\ \;+\beta_{2,1}X_{ijt}+\sigma_{t}+\vartheta_{j}+\mu_{ijt} \end{array}$$

where subscripts *i, j*, and *t* denote respondent, city, and date of completion of the questionnaire, respectively. The *SOC*_*ijt*_ in equation (1) represents the dependent variable, which contains residents' expected month for controlling COVID-19 outbreak at the destination cities, the subjective confidence in controlling COVID-19 at the destination cities, and the sense of confidence [calculated by principal component analysis (PCA)]. *β*_0,1_ denotes the intercept term of the equation (1). The *Wuhan* < *uscore* > *inflow*_*ijt*_ is the independent variable, which represents the population inflow from Wuhan to other cities of China before the Spring Festival. *β*_1,1_ represents regression coefficient of independent variable “*Wuhan* < *uscore* > *inflow*_*ijt*_”. *X*_*ijt*_ denotes the set of control variables as discussed later, and *β*_2,1_ represents regression coefficient of the set of control variables “*X*_*ijt*_”. *σ*_*t*_ and *ϑ*_*j*_ represent the date dummies of completion of the questionnaire and the city dummies of respondents, respectively. Finally, *μ*_*ijt*_ is the error term.

Considering that the residents' expected month for controlling COVID-19 outbreak at the destination cities, and residents' confidence in controlling COVID-19 at the destination cities were measured by ordered variables according to a questionnaire, the ordered probit model (OPM) was employed during this study to estimate equation (1). The ordered probit model is a ranking selection model in which the error distribution follows a standard normal distribution. Meanwhile, given that the variable of the sense of confidence of residents, estimated by PCA, was continuous, ordinary least squares (OLSs) regression was performed to estimate equation (1). OLSs are one of the common methods for estimating model parameters. PCA is a common dimensionality reduction method used in data processing.

Note further information about the questionnaire and the basic characteristics of the respondents are shown in the Supplementary material.

### Data sources and variables selection

Population outflow from Wuhan before the Spring Festival and destination city was the key independent variable of this study. The population inflow from Wuhan to other cities of China before the Spring Festival (January 10th−24th, 2020) was estimated based on open-source indicators from the Baidu Map Migration Big Data Platform and reports from the Wuhan Railway Bureau, Changjiang Net (www.cjn.cn) under the Information Office of Hubei Provincial Government, and Jiemian News (www.jiemian.com) under the Shanghai United Media Group as well as previous inter-region migration data. A 15-day migration dataset was selected from January 10th to 24th, 2020 due to the Spring Festival travel rush beginning on January 10th, after which passenger flow in China remained high. Meanwhile, as a prevention and control measure, Wuhan city, affected by the pandemic, was shut down on January 23rd. However, according to migration data from Baidu, a fraction of the population was observed leaving Wuhan on January 24th. The population outflow from Wuhan was almost zero on January 25th. The volume of passengers traveling by air, railway, and the road was examined. There was a lack of accurate data for the volume of passengers transported by water, however, according to an estimation, passenger volume via this mode of transport was relatively small. An assumption was therefore made that there would be no significant effect on the results due to the absence of volume of passengers transported by water.

Specifically, the Baidu Map Migration Big Data Platform (https://qianxi.baidu.com/) was an indicator of population outflow from Wuhan between January 10th and 24th, 2020, which to some extent, reflected the evolving trend of population outflow from Wuhan before the Spring Festival. Report data and previous inter-region migration data from the Wuhan Railway Bureau, Changjiang Net, and Jiemian News provided outflow population data from Wuhan by railway, air, and road before the Spring Festival. Two estimation methods were adopted to calculate the daily population outflow from Wuhan before the Spring Festival, as described below.

Estimation method one: according to the data reported by Jiemian News, 5,677,625 people left Wuhan by railway, air, and the road between January 10th and 24th, 2020. Based on this figure, the daily population outflow from Wuhan combined with the daily population outflow from Wuhan before the Spring Festival can be estimated via the Baidu Map Migration Big Data Platform. The results of which are shown in column 3 of Table 1.

**Table 1:** Population outflow from Wuhan before the Spring Festival.

**Date**	**Migration scale indicator**	**Outflow by estimation method one (Unit: ten thousand)**	**Outflow by estimation method two (Unit: ten thousand)**	**Mean value (Unit: ten thousand)**
January 10th	6.62	33.86	41.67	37.76
January 11th	7.56	38.67	47.58	43.12
January 12th	6.22	31.81	39.15	35.48
January 13th	5.76	29.46	36.25	32.86
January 14th	5.46	27.93	34.37	31.15
January 15th	5.91	30.23	37.20	33.71
January 16th	6	30.69	37.76	34.23
January 17th	6.44	32.94	40.53	36.74
January 18th	7.71	39.43	48.53	43.98
January 19th	7.41	37.90	46.64	42.27
January 20th	8.31	42.50	52.30	47.40
January 21th	10.74	54.93	67.60	61.26
January 22th	11.84	60.56	74.52	67.54
January 23th	11.14	56.98	70.12	63.55
January 24th	3.89	19.90	24.48	22.19
Total migration from January 10th to 24th (ten thousand)		567.76	698.70	633.23

Estimation method two: according to the data in the report of Changjiang Net, 4.0968 million travel were made by people leaving Wuhan through railway, air, and road from January 10th to 19th, 2020. This data, combined with data on the daily population outflow from Wuhan before the Spring Festival *via* the Baidu Map Migration Big Data Platform, was utilized to estimate the total daily population outflow from Wuhan before the Spring Festival. The results are shown in column 4 of Table 1.

Last, the mean value of the population outflow from Wuhan before the Spring Festival was calculated using method one and method two above. The results of which are presented in column 5 of Table 1. These results demonstrated that the total population outflow from Wuhan was approximately 6 million people. Population outflow peaked 3 days before the implementation of the lock-down measures with daily outflow exceeding 600,000 people. After Wuhan was shut down, the population outflow dropped significantly with only 221,900 people leaving the city on January 24th.

**Figure 1:**
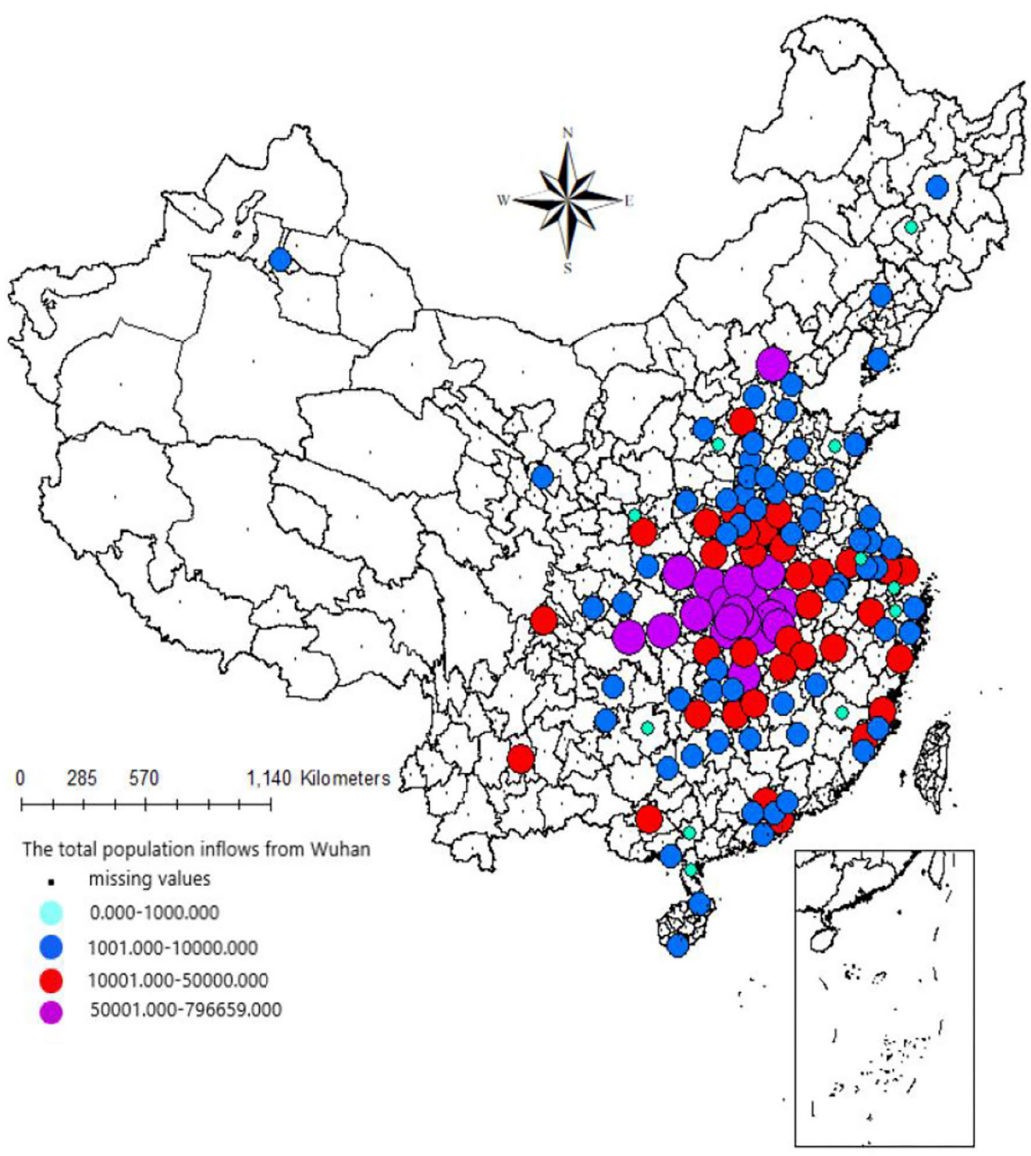
Population inflow from Wuhan city to other cities in China before the Spring Festival. Data source: Baidu Migration Index of China. Available at: http://qianxi.baidu.com

Based on the mean value of the population outflow from Wuhan calculated using the above-mentioned estimation methods, we utilized the percentage indicator of population inflow at various prefecture-level cities in China from January 10th to 24th, 2020 to calculate the daily population inflow from Wuhan to other cities of China. The daily population inflow from Wuhan to other cities of China during the 15 days was summed to obtain the total population inflow at various cities before the Spring Festival. As shown in Figure 1, the results revealed that 70% of the population outflow from Wuhan before the Spring Festival consisted of people who traveled to cities within the Hubei Province. Xiaogan City and Huanggang City had the highest proportions of inflow with 13.84% and 13.15% of the population, respectively. In addition to the cities within the Hubei Province, neighboring cities and provinces received a large proportion of the population inflow from Wuhan. For example, the Xinyang City of the Henan Province and the Changsha City of the Hunan Province had a population inflow of 112,800 people and 81,300 people, respectively. Population inflow from Wuhan was high in four of the first-tier cities in China (the top four cities in mainland China in terms of economic strength), namely Beijing, Shanghai, Guangzhou, and Shenzhen with population inflow numbers of 69,400, 52,600, 39,100, and 38,400 people, respectively.

**Figure 2:**
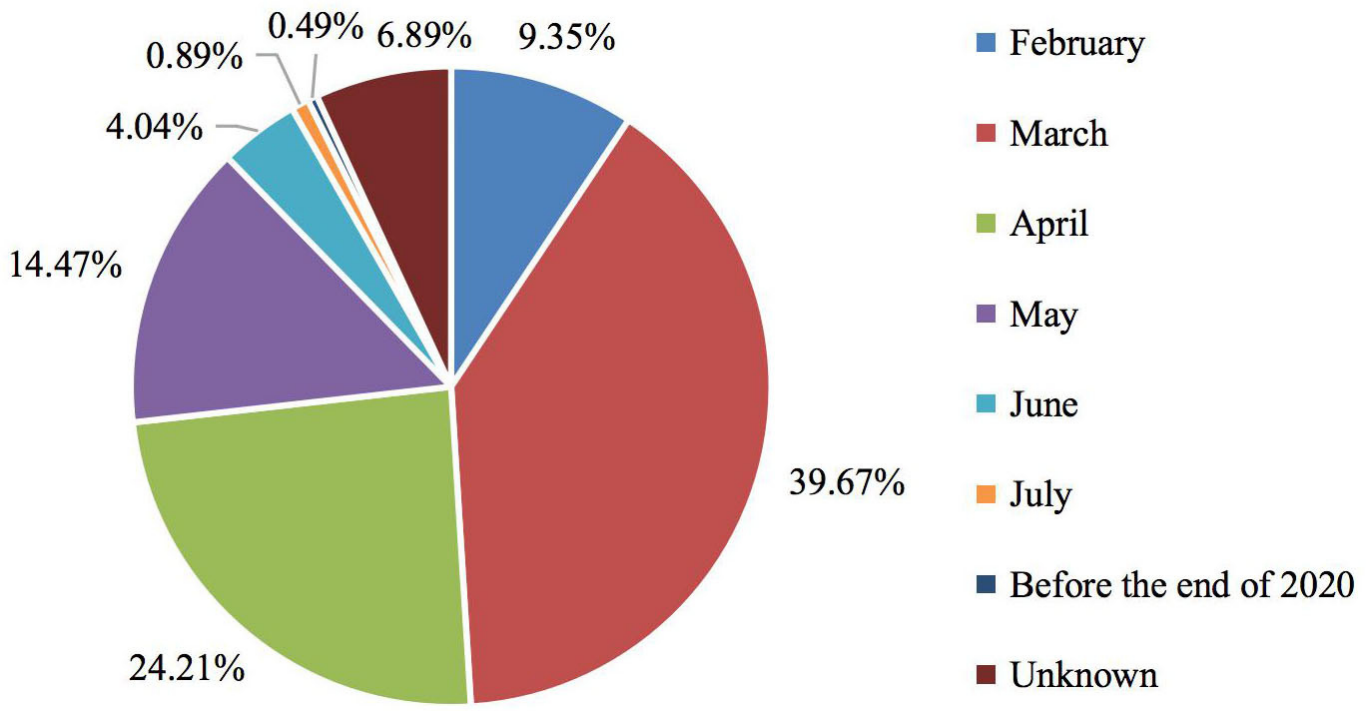
Frequency distribution of residents' expected month for controlling COVID-19 outbreak at the destination cities.

The key dependent variables of this study were residents' expected month for controlling the COVID-19 outbreak at the destination cities and residents' confidence in controlling COVID-19 at the destination cities. Figure 2 presents a frequency distribution showing residents' expected months for controlling the COVID-19 outbreak in the destination cities. 9.35% of respondents thought the disease would be eliminated in February 2020. And 39.67, 24.21, 14.47, and 4.04% of respondents expected the disease would be controlled in March, April, May, and June, respectively. Hence, most residents expected month for control the COVID-19 outbreak in the destination cities during the first half of 2020.

**Figure 3:**
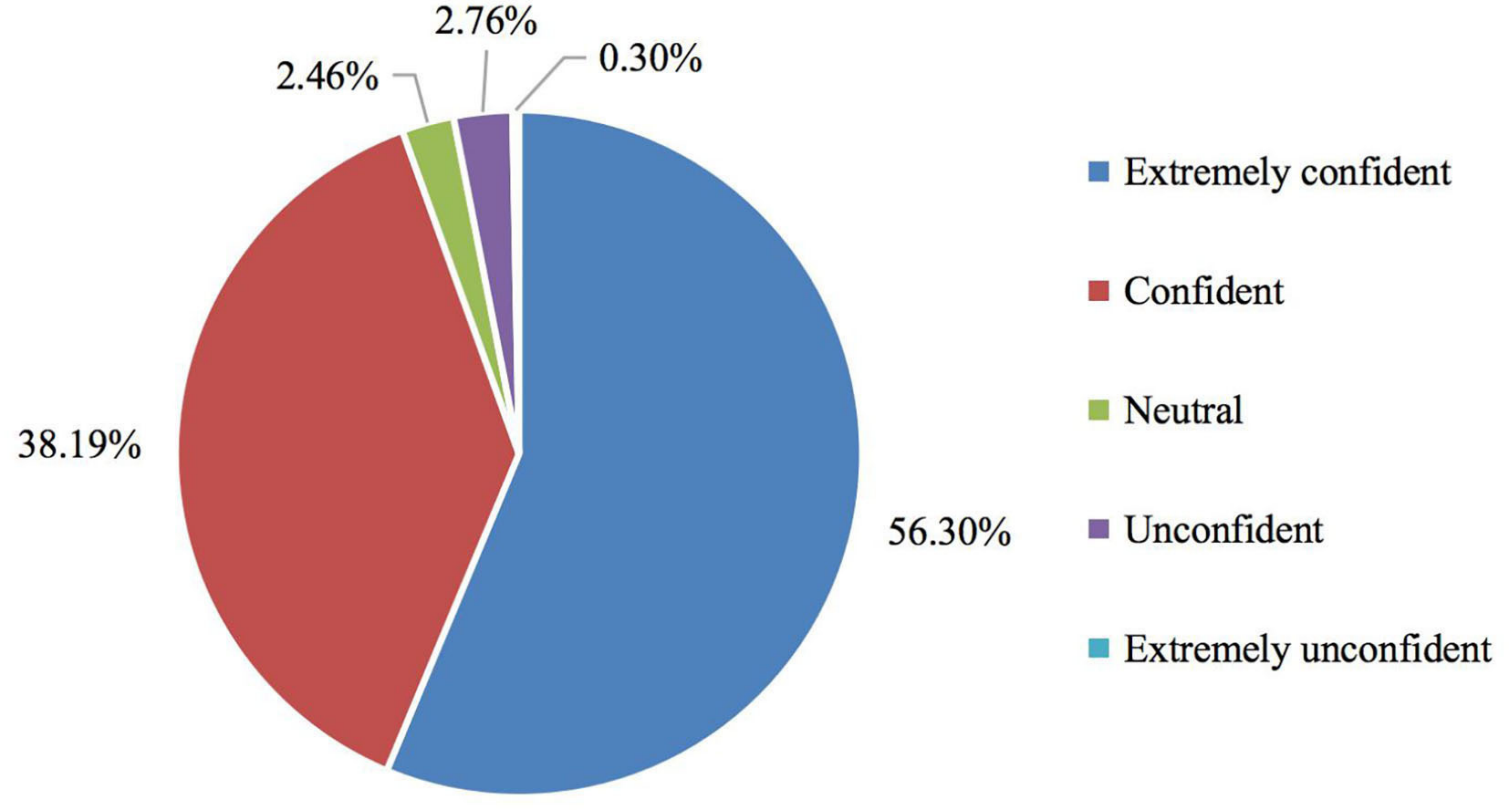
Frequency distribution of residents' confidence in controlling COVID-19 at the destination cities.

The frequency distribution of residents' confidence in controlling COVID-19 at the destination cities, shown in Figure 3, highlighted that most of the respondents were confident that the COVID-19 outbreak would be controlled. Specifically, more than half of respondents (56.30%) were extremely confident and 38.18% of respondents were confident. Approximately 3% of people were unconfident or extremely unconfident that the COVID-19 outbreak would be controlled.

In the empirical model, a large number of factors that can influence residents' expected month for controlling COVID-19 outbreak at the destination cities and residents' confidence in controlling COVID-19 at the destination cities, were also controlled for. These factors included the individual characteristics of the respondents (i.e., gender, age, education level, employment, health status, life difficulty, province of residence, housing location, whether currently living in Hubei province, and housing ownership), community characteristics (i.e., community openness, scale, and occupancy rate), and variables of the COVID-19 outbreak (i.e., news attention related to the novel coronavirus, risk assessment of the novel coronavirus, confirmed cases in the community, suspected cases in the community, quarantine cases in the community, the infection of relatives, friends and colleagues, necessary supplies for pandemic prevention in the community, and the satisfaction of residents with community work regarding disease prevention). The control variable data was derived from the Questionnaire on community and pandemic perception under COVID-19 between February 10th and 25th, 2020. The definitions and descriptive statistics of the key variables are shown in Table 2.

**Table 2:** Definitions and descriptive statistics of key variables.

**Variable name**	**Variable definition**	**Mean**	**Std. Dev**	**Min**	**Max**
Expected month	An ordered variable of residents' expected month for controlling COVID-19 outbreak at the destination cities, which was measured on an eight-point scale where February = 1, March = 2, April = 3, May = 4, June = 5, July = 6, before the end of 2020 = 7, and unknown = 8	3.032	1.720	1	8
Confidence	An ordered variable of the confidence of respondents in eliminating the COVID-19 outbreak, which was measured on a five-point scale where extremely confident = 5, confident = 4, neutral = 3, unconfident = 2, and extremely unconfident=1	4.474	0.707	1	5
SOC	Sense of confidence of respondents in controlling COVID-19 at the destination cities was estimated *via* PCA based on the variables of Confidence and Expected month.	0.000	0.766	−3.594	0.693
Wuhan_inflow	The total population inflow from Wuhan city to other cities of China before the Spring Festival.	33,596	103,162	0	796,659
Satisfaction	The overall satisfaction of the respondents with the community measures for controlling and preventing the COVID-19 outbreak, which was calculated using PCA based on satisfaction with property staff, neighborhood or village committee, owners committee, community health center, and street or township organization.	0.000	1.684	−6.343	2.460
Housing location	An ordered variable of the housing location of respondents was measured on a four-point scale where city center = 4, city suburbs = 3, county or town area = 2, and rural area = 1	2.924	1.190	1	4
Information attention	An ordered variable of the information attention of respondents to the COVID-19 outbreak, which was measured on a five-point scale where very concerned = 5, concerned = 4, generally = 3, not too concerned = 2, and not concerned = 1	4.661	0.582	1	5
Gender	An indicator variable that was equal to one if the respondent was male, and was equal to zero otherwise	0.365	0.482	0	1
Age	An ordered variable of the age of respondents was measured on an eight-point scale were under 12 years old = 1, 12 to 18 years old = 2, 19 to 24 years old = 3, 25 to 35 years old = 4, 36 to 45 years old = 5, 46 to 55 years old = 6, 56 to 65 years old = 7, older than 65 years old = 8	3.814	1.032	2	8
Education	An ordered variable of the education level of respondents was measured on a six-point scale where primary school and below = 1, middle school = 2, senior high school = 3, college or undergraduate = 4, master = 5, and PhD = 6	4.388	0.748	1	6
Housing ownership	An indicator variable of housing ownership, which was equal to one if the respondent was a homeowner, and equal to zero otherwise	0.793	0.405	0	1
Confirmed case	An indicator variable that was equal to one if the community had confirmed cases of COVID-19, and equal to zero otherwise	0.057	0.232	0	1
Suspected case	An indicator variable that was equal to one if the community had suspected cases of COVID-19, and equal to zero otherwise	0.033	0.180	0	1
Quarantine case	An indicator variable that was equal to one if the community had quarantine cases of COVID-19, and equal to zero otherwise	0.147	0.354	0	1
Supply	An ordered variable measured on a four-point scale which represented the supply of the goods in nearby pharmacies, hospitals, supermarkets, these goods were related to the COVID-19 prevention where available = 4, basically available = 3, basically unavailable = 2, and unavailable = 1	2.278	0.689	1	4
Community openness	An ordered variable of the community openness in peacetime, which was measured on a three-point scale where closed wall management = 1, open wall management = 2, and totally open = 3	1.907	0.929	1	4
Community scale	An ordered variable of the community scale, which was measured on a five-point scale where <100 households=1, 100 to 500 households = 2, 500–1,000 households = 3, 1,000–2,000 households = 4, and more than 2,000 households = 5	2.634	1.195	1	5
Community occupancy	An ordered variable of the community occupancy rate during the COVID-19 prevention period compared to that in peacetime where much lower = 1, similar = 2, much more = 3	1.854	0.653	1	3
Other case	An indicator variable that was equal to one if there were relatives, friends or colleagues infected with COVID-19, and equal to zero otherwise	0.960	0.197	0	1
Risk assessment	An ordered variable of self-assessed COVID-19 risk where extremely high = 5, high = 4, moderate = 3, low = 2, extremely low = 1	3.797	0.962	1	5
Health status	An ordered variable of self-assessed health status where extremely healthy = 5, healthy = 4, moderately healthy = 3, unhealthy = 2, extremely unhealthy = 1	4.629	0.545	2	5
Life difficulty	An indicator variable that was equal to one if respondents were facing life difficulties during the survey period, and equal to zero otherwise	0.643	0.479	0	1
Employed	An indicator variable that was equal to one if respondents were employed, and equal to zero otherwise	0.498	0.500	0	1
Hubei resident	An indicator variable that was equal to one if respondents were living in Hubei province, and equal to zero otherwise	0.061	0.239	0	1

## Results

In this section, the relationship between population inflow from Wuhan city and the sense of confidence of residents in controlling the COVID-19 outbreak at the destination cities was investigated. Specifically, the basic findings, including the impact of population inflow from Wuhan city on residents' expected month for controlling COVID-19 outbreak at the destination cities and residents' confidence in controlling COVID-19 at the destination cities, are presented. Additionally, robustness checks were conducted to ensure the credibility of the empirical findings. Lastly, marginal effect analysis was conducted to obtain more valuable information on basic relationships.

### Benchmark results

Data presented in Table 3 show the impact of population inflow from Wuhan city on the residents' expected month for controlling COVID-19 outbreak at the destination cities. Table 3 also contains the estimated coefficients, robust clustered standard errors, and significance levels for the key independent variables. Logarithmic population inflow from Wuhan city, and city and date fixed effects were controlled for. The coefficient in column 1 (Table 3) indicates that without controlling for any other factors (i.e., individual characteristics, community characteristics, and the variables of COVID-19), the population inflow from Wuhan city significantly extended the month that residents in the destination cities expected COVID-19 would be controlled. That is, the more people that left Wuhan city before the lock-down was implemented, the longer the disease was expected to last. In specification 2 the individual characteristics of the respondents (i.e., gender, age, education level, employment, health status, life difficulty, province of residence, housing location, whether currently living in Hubei province, and housing ownership) were controlled for, and the results (Table 3, column 2) remained positive and were significant at the 1% level. In specification 3, community characteristics (i.e., community openness, scale, and occupancy rate) were controlled for (Table 3, column 3), and the results were also significantly positive. The variables of the COVID-19 outbreak (i.e., news attention related to the novel coronavirus, risk assessment of the novel coronavirus, confirmed cases in the community, suspected cases in the community, quarantine cases in the community, the infection of relatives, friends, and colleagues, necessary supplies for pandemic prevention in the community, and the satisfaction of residents with community work about disease prevention) were also controlled for, and the results (Table 3, column 4) were also positive and significant at the 1% level. The consistency of these results indicated that the population inflow from Wuhan prolonged residents' expected month for controlling the COVID-19 outbreak at the destination cities. Therefore, the results presented in Table 3 demonstrate the importance of the temporary closure of Wuhan for the early defeat of the COVID-19 outbreak, from the subjective expectations of the residents.

**Table 3:** The impact of population inflow from Wuhan city on residents' expected month for controlling COVID-19 outbreak at the destination cities.

	**(1)**	**(2)**	**(3)**	**(4)**
	**Dependent variable: residents' expected month for controlling COVID-19 outbreak at the destination cities**			
	**OPM**	**OPM**	**OPM**	**OPM**
Independent variable				
Ln(Wuhan_inflow)	0.792^***^	0.792^***^	0.838^***^	0.779^***^
	(0.029)	(0.031)	(0.031)	(0.035)
Control variable				
Individual characteristics	No	Yes	Yes	Yes
Community characteristics	No	No	Yes	Yes
Variables of COVID-19	No	No	No	Yes
City dummies	Yes	Yes	Yes	Yes
Date dummies	Yes	Yes	Yes	Yes
Observations	1,016	1,016	1,016	1,016

*Clustered standard errors in parentheses;*

* *p < 0.1*,

** *p < 0.05*,

*** *p < 0.01*.

The relationship between population inflow from Wuhan city and residents' confidence in controlling the COVID-19 outbreak at the destination cities was also tested. As shown in Table 4, the control variables were gradually increased (from columns 1 to 4). The estimated coefficients in each column remained negative and were all significant at the 1% level, suggesting that the population inflow from Wuhan city lowered residents' confidence in the destination cities in overcoming the COVID-19 outbreak. Again, from the standpoint of residents' subjective confidence, the empirical result from Table 4 also tells the significance of Wuhan's temporary closure for controlling COVID-19.

**Table 4:** The impact of inflow of people from Wuhan city on residents' confidence in controlling COVID-19 outbreak at the destination cities.

	**(1)**	**(2)**	**(3)**	**(4)**
	**Dependent variable: residents' confidence in controlling COVID-19 at the destination cities**			
	**OPM**	**OPM**	**OPM**	**OPM**
Independent variable				
Ln(Wuhan_inflow)	−0.705^***^	−0.701^***^	−0.708^***^	−0.677^***^
	(0.026)	(0.030)	(0.032)	(0.034)
Control variable				
Individual characteristics	No	Yes	Yes	Yes
Community characteristics	No	No	Yes	Yes
Variables of COVID-19	No	No	No	Yes
City dummies	Yes	Yes	Yes	Yes
Date dummies	Yes	Yes	Yes	Yes
Observations	1,016	1,016	1,016	1,016

*clustered standard errors in parentheses;*

* *< 0.1*,

** *p < 0.05*,

*** *p < 0.01*.

Based on the overall indicators of the sense of confidence of residents in controlling the COVID-19 outbreak at the destination cities (calculated *via* PCA), the effect of population inflow from Wuhan city on the sense of confidence of residents in controlling the COVID-19 outbreak at the destination cities was examined. The results are presented in Table 5. The coefficient in column 1 (-0.100) was significant at the 1% level before controlling for other variables. After gradually controlling for individual characteristics, community characteristics and variables of COVID-19 (columns 2 to 4), the coefficients were also statistically negative at the 1% level. This indicated that the population inflow from Wuhan city significantly reduced the sense of confidence of residents in controlling the COVID-19 outbreak in the destination cities. Therefore, the temporary closure of Wuhan was a critical measure in the control and prevention of the spread of COVID-19. Additionally, the results demonstrate that the closure of Wuhan also strengthened the sense of confidence of residents.

**Table 5:** The impact of population inflow from Wuhan city on the sense of confidence of residents in controlling COVID-19 outbreak at the destination cities.

	**(1)**	**(2)**	**(3)**	**(4)**
	**Dependent variable: Overall SOC**			
	**OLS**	**OLS**	**OLS**	**OLS**
Independent variable				
Ln(Wuhan_inflow)	−0.100^***^	−0.095^***^	−0.102^***^	−0.072^***^
	(0.008)	(0.012)	(0.014)	(0.016)
Control variable				
Individual characteristics	No	Yes	Yes	Yes
Community characteristics	No	No	Yes	Yes
Variables of COVID-19	No	No	No	Yes
City dummies	Yes	Yes	Yes	Yes
Date dummies	Yes	Yes	Yes	Yes
Observations	1016	1016	1016	1016

*Clustered standard errors in parentheses;*

* *p < 0.1*,

** *p < 0.05*,

*** *p < 0.01*.

### Robustness checks

To make the results from Tables 3, 5 more convincing, robustness checks were conducted on benchmark regression results including changing the sample range and replacing measurement indicators of the population inflow from Wuhan city to other cities in China. Considering that the severity of COVID-19 in the Hubei province in central China was at a peak when this survey was conducted, it was important to remove the potential effects of extreme values and re-estimate equation (1). Table 6 shows the robustness check results based on the new sample without observations from Hubei province. The independent variables in columns 1 and 2 (Table 6) are the months that residents expected COVID-19 would be controlled. The coefficients were positive and were all statistically significant at the 1% level, indicating that the population inflow from Wuhan city extended residents' expected month for controlling the COVID-19 outbreak at the destination cities. These findings corroborated the basic findings presented in Table 3. Meanwhile, as shown in columns (3) and (4), the impact of population inflow from Wuhan city on residents' confidence in controlling the COVID-19 outbreak at the destination cities was negative at the 1% level, the results again suggest that the as for the residents at the destination cities, the population inflow from Wuhan city could significantly weaken their confidence, and it is also consistent with the basic finding in Table 4. More importantly, the coefficients in columns 5 and 6 remained significantly negative regardless of whether other variables were controlled for, revealing the population inflow from Wuhan city was disadvantageous for the sense of confidence of residents. These results are also consistent with the findings presented in Table 5. Overall, after removing the sample from Hubei province, the benchmark regression results are robust.

**Table 6:** Excluding the sample from Hubei province.

	**(1)**	**(2)**	**(3)**	**(4)**	**(5)**	**(6)**
	**Dependent variable: the sense of confidence of residents in controlling COVID-19 at the destination cities**					
	**Expected month**		**Confidence**		**Overall SOC**	
	**OPM**	**OPM**	**OPM**	**OPM**	**OLS**	**OLS**
Independent variable						
Ln(Wuhan_inflow)	0.792^***^	0.809^***^	−0.699^***^	−0.664^***^	−0.098^***^	−0.068^***^
	(0.029)	(0.036)	(0.026)	(0.035)	(0.008)	(0.017)
Control variable						
Individual characteristics	No	Yes	No	Yes	No	Yes
Community characteristics	No	Yes	No	Yes	No	Yes
Variables of COVID-19	No	Yes	No	Yes	No	Yes
City dummies	Yes	Yes	Yes	Yes	Yes	Yes
Date dummies	Yes	Yes	Yes	Yes	Yes	Yes
Observations	954	954	954	954	954	954

clustered standard errors in parentheses; *p < 0.1, **p < 0.05,

*** *p < 0.01*.

Independent variables were further replaced with the population density inflow from Wuhan city to other cities in China. That is, the new independent variable for the robustness check was the population inflow from Wuhan city divided by the total registration population of the destination cities elsewhere at the end of 2017. The data for the total registration population at the end of 2017 was collected from the National Statistical Yearbook of China which was published by the National Bureau of Statistics of China^1^ The total registration population at the end of 2017 was chosen as the denominator due to the availability of data, and the negligible change in population trends during that time. Table 7 shows the results of robustness checks by replacing the independent variable. The results show that the population inflow from Wuhan city was positively correlated with residents' expected month for controlling COVID-19 outbreak at the destination cities and that it also negatively affected residents' confidence in controlling COVID-19 at the destination cities. The coefficients in columns 5 and 6 (Table 7) were negative and were statistically significant at the 1% level, which suggests that blocking the population inflow from Wuhan depressed the sense of confidence of residents in controlling the COVID-19 outbreak at the destination cities. Therefore, the results of the robustness check again proved that the previous basic findings were robust and credible.

**Table 7:** Replacement of the independent variable with density.

	**(1)**	**(2)**	**(3)**	**(4)**	**(5)**	**(6)**
	**Dependent variable: the sense of confidence of residents in controlling COVID-19 at the destination cities**					
	**Expected month**		**Confidence**		**Overall SOC**	
	**OPM**	**OPM**	**OPM**	**OPM**	**OLS**	**OLS**
Independent variable						
Density (every 10 thousand people)	0.058^***^	0.057^***^	−0.053^***^	−0.048^***^	−0.010^***^	−0.008^***^
	(0.002)	(0.003)	(0.003)	(0.003)	(0.000)	(0.001)
Control variable						
Individual characteristics	No	Yes	No	Yes	No	Yes
Community characteristics	No	Yes	No	Yes	No	Yes
Variables of COVID-19	No	Yes	No	Yes	No	Yes
City dummies	Yes	Yes	Yes	Yes	Yes	Yes
Date dummies	Yes	Yes	Yes	Yes	Yes	Yes
Observations	1,016	1,016	1,016	1,016	1,016	1,016

clustered standard errors in parentheses; *p < 0.1, **p < 0.05,

*** *p < 0.01*.

### Marginal effect analysis

Considering that the meanings of the coefficients estimated by the OPM were not intuitive, they could only provide the signs and significance levels for the key independent variable. Thus, marginal effect analysis was conducted to obtain more valuable information on the basic results. Additionally, an attempt was made to calculate the probability change in the dependent variable with per unit change in the explanatory variable when all other control variables were at the mean. Given that there were two discrete dependent variables in this study, including the residents' expected month for controlling COVID-19 outbreak at the destination cities, and residents' confidence in controlling COVID-19 at the destination cities, two equations (equation 2 and equation 3) were constructed to estimate the marginal effect of population inflow from Wuhan city on the two above-mentioned dependent variables, as follows:


(2) $$\begin{array}{l} ME\;(Expected\;month)=\frac{\partial \;Prob(Expected\;month\;=m)}{\partial \;Ln\;(Wuhan\_info\;low)}{|}_{x=\bar{x}}\;\\ \;(m=1,2,3,4,5,6,7,8)\; \end{array}$$

(3) $$\begin{array}{l} ME\;(Confidence)=\frac{\partial \;Prob(Confidence=n)}{\partial \;Ln\;(Wuhan\_info\;low)}{|}_{x=\bar{x}}\\ \;(n=1,2,3,4,5) \end{array}$$

where the variables in equations 2 and 3 are defined in Table 1. The values of expected month ranged from 1 to 8, and corresponded with the expected months that the COVID-19 outbreak would be controlled which were “February,” “March,” “April,” “May,” “June,” “July,” “before the end of 2020,” and “unknown,” respectively. The values of Confidence ranged from 1 to 5, and corresponded to the subjective confidence of people in combating COVID-19 which were “extremely unconfident,” “unconfident,” “neutral,” “confident,” and “extremely confident,” respectively.

Table 8 presents the results on the marginal effects of population inflow from Wuhan city on residents' expected month for controlling COVID-19 outbreak at the destination cities. The data shows that when the population density inflow from Wuhan city increased by one additional unit, the probabilities of “February” and “March” significantly decreased by 0.1023, and 0.1602, respectively, while the probabilities of “April,” “May,” “June,” “July,” “before the end of 2020,” and “unknown” significantly increased by 0.0470, 0.0856, 0.0333, 0.0080, 0.0046, and 0.0840, respectively. Therefore, the higher the population inflow from Wuhan city, the longer the time that the residents in the destination cities perceived the COVID-19 outbreak would last. This also confirms the significance of the temporary closure of Wuhan during the COVID-19 outbreak.

**Table 8:** Marginal effect analysis of the residents' expected month for controlling COVID-19 outbreak at the destination cities.

**Expected**	**Marginal**	**Delta-method**	**Significance**
**months**	**effect**	**std. err**.	**level**
February	−0.1023	0.0072	***
March	−0.1602	0.0104	***
April	0.0470	0.0059	***
May	0.0856	0.0074	***
June	0.0333	0.0053	***
July	0.0080	0.0025	***
Before the end of 2020	0.0046	0.0022	**
Unknown	0.0840	0.0093	***

*p < 0.1, **p < 0.05, ***p < 0.01.

Moreover, Table 9 shows the results of the marginal effects of population inflow from Wuhan city on residents' confidence in controlling the COVID-19 outbreak in the destination cities. as shown in Table 9, when the population inflow from Wuhan city increased by one additional unit, the probability of ‘extremely confident' of residents in the destination cities decreased by 0.1973; the probabilities of “confident,” “neutral,” and “unconfident” of residents in the destination cities significantly increased by 0.1392, 0.0224, and 0.0320, respectively; while the probability changes of ‘extremely unconfident' of residents in the destination cities was insignificant. Hence, the study results highlight the importance of ceasing the population outflow from Wuhan city from the standpoint of the subjective confidence of residents in controlling COVID-19 in the destination cities. This is also consistent with the findings presented in Table 8.

**Table 9:** Marginal effect analysis for confidence in controlling COVID-19 at the destination cities.

**Expected**	**Marginal**	**Delta-method**	**Significance**
**months**	**effect**	**std. err**.	**level**
Extremely confident	−0.1973	0.0113	***
Confident	0.1392	0.0096	***
Neutral	0.0224	0.0036	***
Unconfident	0.0320	0.0047	***
Extremely unconfident	0.0037	0.0024	

*p < 0.1, **p < 0.05, ***p < 0.01.

## Conclusion and discussion

As the COVID-19 outbreak spreads throughout China and across the globe, fear of the pandemic is also spreading. The confidence of people in an early victory over the disease directly affects their morale, which in turn affects the effectiveness of disease prevention and the overall stability of society. Hence, from the perspective of the sense of confidence of residents in controlling COVID-19 in the destination cities, this study attempted to provide evidence for the significance of the temporary closure of Wuhan city in China. Based on the data from the Questionnaire on community and pandemic perception under COVID-19 and estimates of population inflow from Wuhan to the rest of the country before the Spring Festival, we employ a multiple regression model to examine the impact of the population inflow from Wuhan city on residents' expected month for controlling COVID-19 outbreak at the destination cities, residents' confidence in controlling COVID-19 outbreak at the destination cities, and the overall indicators for the sense of confidence of residents in controlling COVID-19. Moreover, we also employ a marginal effect analysis to calculate the probability of change in residents' confidence in controlling the COVID-19 outbreak with per unit change in the population inflow from Wuhan city.

First, benchmark result shows that in controlling for the individual characteristics of respondents, community characteristics, and variables of the COVID-19 outbreak, the impact of population inflow from Wuhan city on residents' expected month for controlling COVID-19 outbreak at the destination cities was positive and significant at the 1% level. Robustness checks conducted on benchmark regression results, including changing the sample range, and replacing measurement indicators of the population inflow from Wuhan city, demonstrated that the basic findings were robust and credible. Marginal effect analysis shows that when the population inflow from Wuhan city increased by one additional unit, the probabilities of the variables “February” and “March” decreased significantly by 0.1023 and 0.1602, respectively, while the probabilities of “April,” “May,” “June,” “July,” “before the end of 2020,” and “unknown” significantly increased by 0.0470, 0.0856, 0.0333, 0.0080, 0.0046, and 0.0840, respectively. This indicates that the population inflow from Wuhan prolonged the residents' expected month for controlling the COVID-19 outbreak at the destination cities.

Second, the benchmark result shows that the impacts of population inflow from Wuhan on residents' confidence in controlling the COVID-19 outbreak at the destination cities were negative and significant at the 1% level. Robustness checks conducted on benchmark regression results, including changing the sample range, and replacing measurement indicators of the population inflow from Wuhan city, demonstrated that these findings were robust and credible. Marginal effect analysis shows that when the population inflow from Wuhan city increased by one additional unit, the probability of the variable “extremely confident” decreased by 0.1973. Furthermore, the probabilities of the variables “confident,” “neutral,” and “unconfident” significantly increased by 0.1392, 0.0224, and 0.0320, respectively. This suggests that the population inflow from Wuhan city lowered residents' confidence in controlling COVID-19 in the destination cities.

Finally, based on the overall indicators for the sense of confidence of residents in controlling COVID-19 outbreak at the destination cities (calculated *via* PCA), the effect of population inflow from Wuhan city on the sense of confidence of residents in controlling COVID-19 outbreak at the destination cities was examined. The result shows that the impacts of population inflow from Wuhan on the sense of confidence of residents in controlling the COVID-19 outbreak at the destination cities were negative and significant at the 1% level. Robustness checks conducted on benchmark regression results, including changing the sample range, and replacing measurement indicators of the population inflow from Wuhan city, demonstrated that previous basic findings were robust and credible. This indicates that the population inflow from Wuhan significantly lowered the sense of confidence of residents in controlling the COVID-19 outbreak in the destination cities.

In summary, we find that the population inflow from Wuhan city played a negative role in the sense of confidence of residents in controlling COVID-19 in the destination cities. The higher the population inflow from Wuhan city, the longer is the residents' expected month for controlling COVID-19 outbreak at the destination cities, and weaker the residents' confidence in controlling COVID-19 outbreak at the destination cities. The results of this study indicate that in most plausible outbreak scenarios, the temporary closure of Wuhan city contributed to the sense of confidence of residents in controlling the COVID-19 outbreak in the destination cities. Such measures can also aid in improving the optimism and expectations of residents living in cities outside the outbreak area.

## Data availability statement

The raw data supporting the conclusions of this article will be made available by the authors, without undue reservation.

## Author contributions

XG, YW, and SZ wrote the original draft. ZW and YZ prepared Figures 1–3. XG, ZW, YW, YZ, and SZ revised the original draft. All authors reviewed the manuscript. All authors contributed to the article and approved the submitted version.

## Conflict of interest

The authors declare that the research was conducted in the absence of any commercial or financial relationships that could be construed as a potential conflict of interest.

## Publisher's note

All claims expressed in this article are solely those of the authors and do not necessarily represent those of their affiliated organizations, or those of the publisher, the editors and the reviewers. Any product that may be evaluated in this article, or claim that may be made by its manufacturer, is not guaranteed or endorsed by the publisher.

## Footnotes

^1^Available at: http://www.stats.gov.cn.
