# Supplementary material for: The impact of lockdown in Wuhan on residents confidence in controlling COVID-19 outbreak at the destination cities
Source: Front Public Health. 2022 Aug 15;10:902455. doi: 10.3389/fpubh.2022.902455 (PMC9421152; doi:10.3389/fpubh.2022.902455)
Supplement: Supplementary file 1 [file Data_Sheet_1.pdf]

Supplementary Material

1 The basic characteristics of the respondents

The relevant options for the questionnaires covered in this paper are described in Table 2 of our original manuscript. We further describe the regional distribution, housing location, gender, age and education level of the respondents as follows.

1.1 Regional distribution of the respondents

The data collected covered 31 provinces, municipalities, and autonomous regions in mainland China, as well as the Hong Kong Special Administrative Region, and Taiwan. A total of 1,060 questionnaires were distributed, of which 1,049 valid questionnaires were obtained and 9.06% of those were collected from the hardest-hit Hubei Province. The percentage of provinces with less than 1% of respondents was 3.89%, including Liaoning, Gansu, Inner Mongolia, Ningxia, Hong Kong and Taiwan.

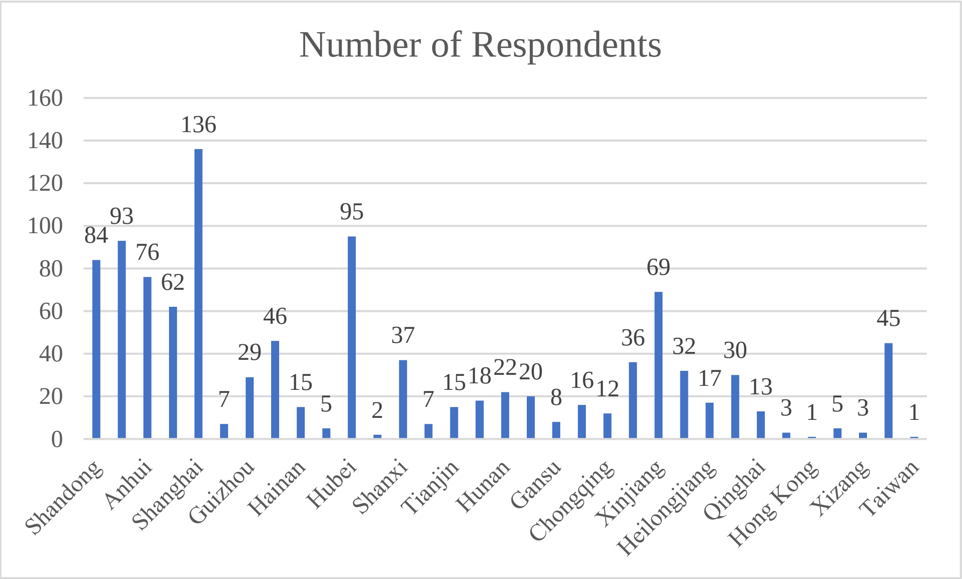

1.2. Housing location of the respondents

According to the area in which the respondents currently live, the distribution of respondents' areas is relatively balanced between urban and rural areas. 49.29% of respondents live in urban centres, while around half live in rural areas, county townships and suburban areas.

Housing location	Number of Respondents	Percentage
**A. Urban centres**	**523**	**49.34%**
**B. Suburban areas**	**152**	**14.34%**
**C. County townships**	**188**	**17.74%**
**D. Rural areas**	**197**	**18.58%**

1.3. Gender of the respondents

In terms of gender representation, there were more women than men among the respondents.

Gender	Number of Respondents	Percentage
**A. Male**	**385**	**36.32%**
**B. Female**	**675**	**63.68%**

1.4. Age range of the respondents

The age range of respondents was mainly in the 19-55 range, with the highest percentage of respondents in the 19-24 range at 41.6% and 38.21% in the 25-35 age range.

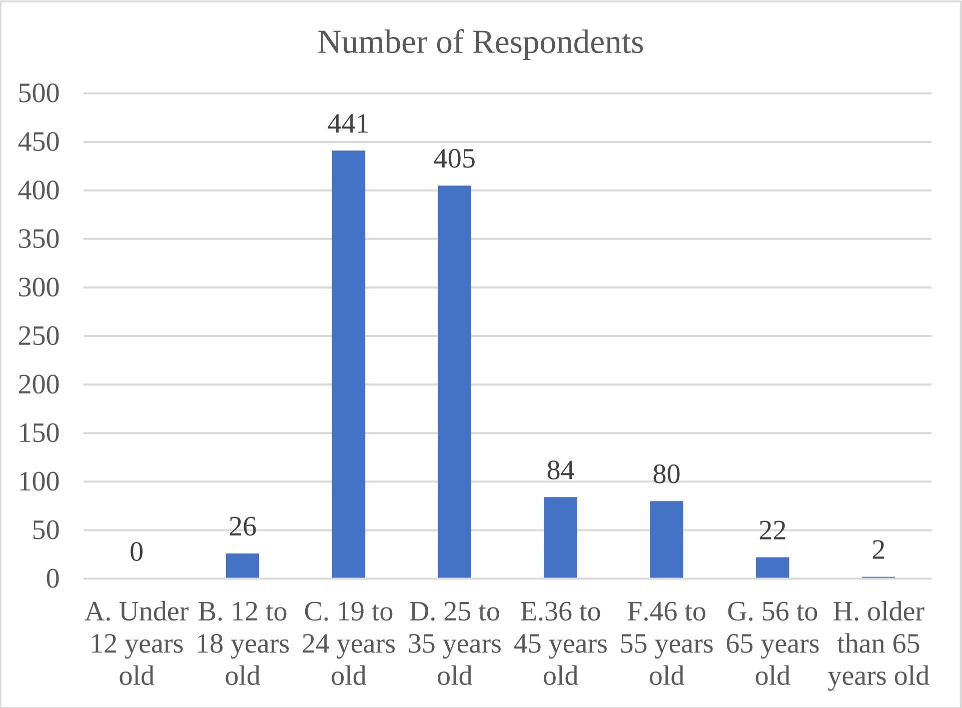

1.5. Education level of the respondents

The education level of the respondents is mainly college and master, with more than half of them having a College's degree (55.38%) and 32.45% having a master's degree.

Education level	Number of Respondents	Percentage
A. Primary school and below	1	0.09%
B. Middle school	18	1.70%
C. Senior high school	35	3.30%
D. College or undergraduate	587	55.38%
E. Master	344	32.45%
F. PhD	75	7.08%

2 Questionnaire on community and pandemic perception under COVID-19

Dear friends,

Hello! In order to strengthen the scientific effectiveness of COVID-19 prevention and control, and to better serve the relevant government departments in COVID-19 prevention and control, the Center for Housing and Urban-Rural Development of *Shanghai Jiao Tong University* is organising an online survey on the relationship between community and epidemic awareness. The survey will only take about 6-8 minutes to complete, so we ask for your cooperation and careful and truthful input. All information will be used for research and policy recommendations only and will not be disseminated.

Thank you for your support!

1. You are currently living in ______ province _______ city

D. Rural area

3. Are you very concerned about the information on the COVID-19 outbreak?
A. Very concerned; B. Concerned; C. Generally concerned; D. Not too concerned; E. Not concerned

4. What is your gender?
A. Male; B. Female

5. What is your age group?
A. Under 12 years old; B. 12 to 18 years old; C. 19 to 24 years old; D. 25 to 35 years old; E. 36 to 45 years old; F. 46 to 55 years old; G. 56 to 65 years old; H. older than 65 years old

6. What is your education level?
A. Primary school and below; B. Middle school; C. Senior high school; D. College or undergraduate; E. Master; F. PhD

7. Have you and any members of your family travelled overseas after 20 January?
A. No; B. Yes, traveled outside or abroad or back home (not to Hubei); C. Yes, visited Hubei

8. What is your current residence and state of residence?
A. Permanent residence with free movement or voluntary isolation; B. Permanent residence with compulsory isolation; C. Home town with free movement or voluntary isolation; D. Home town with compulsory isolation; E. Temporary residence during return or journey; F. Residence in non-residential place of permanent residence; G. Compulsory isolation in non-residential place; H. Under medical treatment in hospital; I. Other _________

9. What is the ownership status of your current residence?
A. Self-purchased commercial housing; B. Purchased public housing; C. Relocation compensation housing; D. Property ownership housing (affordable housing, shared ownership housing, etc.); E. Self-built housing; F. Borrowing from friends and relatives; G. Rental housing provided by the unit; H. Housing rented directly from a private landlord; I. Housing rented from a professional agency, long term rental flat or similar type; J. Rental housing provided by the government; K. Not sure; L. Others _________

10. Which of the following people, other than you, live with you in your current home?

	My spouse/partner	Children aged 0-6	Teenagers aged 6-18	College students	Parents or spouse's parents	Grandparents or other relatives	Non-relative members
A. Yes							
B. No							

11. How do you rate the hygiene of your current home and community living environment on a regular basis?

	Very clean	Clean	Generally	Not clean	Very unclean	Unable to rate
A. Residence						
B. Neighbourhood (village)						
C. Street (town)						

12. How do you rate the property management company, residents' committee and owners committee in your community?

	Very good	Good	Generally	Bad	Very bad	Not sure
A. Property management company						
B. Residents' committee						
C. Owners committee						

13. How convenient is the community you currently live in in general?

	Very convenient	Convenient	Generally	Inconvenient	Very inconvenient	Not sure
A. Shopping						
B. Buying medicine						
C. Going to the doctor						
D. Fitness						
E.Transportation						
F. Entertainment						

14. How would you rate the measures taken by the organisations and units during the COVID-19 prevention period ?

	Very satisfactory	Satisfactory	Generally	Unsatisfactory	Very unsatisfactory	Unable to rate

A. Property management company						
B. Residents' committee						
C. Owners committee						
D.Community Health Centres						
E.Street/township institutions						

15. As far as you know, what are the main preventive and control measures taken by your community in response to the COVID-19 outbreak (multiple answers allowed)?
A. Provide a community group to immediately disseminate information on the prevention and control of the epidemic; B. Take body temperature in and out of the community; C. Require the wearing of masks in and out of the community; D. Strictly control entrances and exits of the community; E. Strictly prohibit non-community people, including couriers, entering the community; F. Visit and investigate; G. Hang relevant banners and distribute brochures and leaflets; H. Strengthen the cleaning and disinfection of lifts, buildings, roads, etc.; I. Assist the government in registration and distribution of prevention and control items such as masks; J. Strictly isolate suspected persons and seal off relevant areas; K. Only one family member per household can enter or leave every few days; L. Others ________________

16. In view of this epidemic, what other aspects of the COVID-19 prevention work in your community do you think need to be strengthened (multiple answers allowed)?
A. Control of entrances and exits in the community; B. Publicity, persuasion and disinformation for community residents; C. Management of community outsiders such as couriers; D. Monitoring and health management of focus groups; E. Care of vulnerable people in the community (e.g. elderly people living alone, children left behind, low-income families, families with major diseases, etc.); F. Distribution and allocation of epidemic prevention materials; G. Management of the community environmental sanitation; H. Management of public spaces in the community (management of gathering risk points); I. Psychological safety promotion and guidance for residents; J. Manpower input for community management service personnel; K. Others ________________

17. (Tenants only) As a tenant, do you think there is a differential approach for tenants in your community in terms of the COVID-19 prevention (if you do not choose A, you can choose more than one of the other options)?
A. No; B. Yes, access to the community is more difficult than for landlords; C. Yes, access to epidemic prevention items is more difficult than for landlords; D. Yes, community workers are less concerned and helpful; E. Yes, other ________________

18. Up to now, how often do you go in and out of your neighbourhood/village during the COVID-19 prevention period?

A. Two to three times a day; B. Once a day; C. Once every two or three days; D. Once a week; E. Once every fortnight; F. Haven't been out for a fortnight

19. Do you understand and support the inconvenience caused by the COVID-19 prevention and control measures?
A. Very much; B. More understanding and supportive; C. Moderately; D. Not very much, but I can only comply; E. Very little, not necessary at all

20. As far as you know, are there any confirmed cases, suspected cases or isolated patients of the COVID-19 in your community?
A. There are confirmed cases; B. No confirmed cases, but there are suspected patients; C. No confirmed or suspected cases, but there are isolated patients; D. None of them; E. Not sure

21. As far as you know, are basic COVID-19 prevention items (e.g. masks, alcohol disinfectant, over-the-counter medicines, etc.) currently available at pharmacies, hospitals and supermarkets in the vicinity of this community?
A. Yes; B. Some shortages; C. No; D. Not sure

22. Apart from the inconvenience of going out and shopping, do you have any difficulties in living at present? (Multiple choice possible)
A. No difficulties, living materials are secure; B. There are many shortages of living materials, not easy to buy, otherwise fine; C. Living materials are basically secure, but worried about the risk of unemployment or reduced income; D. There is a serious shortage of living materials, difficult to buy, and worried about the risk of unemployment or reduced income; E. Someone in the family is in poor health or has a chronic disease, it is difficult to see a doctor; F. Other _______________

23. Have the cadres or community workers in your community given you and your family any support and help in life?
A. A lot, solving a lot of problems; B. Some, solving some problems; C. Some, but not solving problems; D. No help at all; E. Not only no help, but also creating a lot of difficulties; G. Other
____

24. As far as you know, what is the situation of the type of community you live in now?
A. New community (built after 2010); B. Second new community (built between 2000 and 2010); C. Older community (built between 1990 and 2000); D. Older community (built before 1990); E. Unit dormitory area; F. Urban village; G. Rural

25. As far as you know, the community where you live now is normally a:
A. Closed wall management; B. Open wall management; C. Totally open; D. other ______

26. As far as you know, what is the approximate size of your neighbourhood/village, if it is full?

A. less than 100 households; B. 100 to 500 households; C. 500 to 1000 households; D. 1000 to 2000 households; E. more than 2000 households

27. According to your estimate, how does the current occupancy rate in your neighbourhood/village compare with the usual rate?
A. Much lower than usual; B. About the same as usual; C. Higher than usual

28. Has a friend or relative close to you been confirmed or suspected of COVID-19? (Multiple answers possible)
A. A close relative has been confirmed; B. A distant relative has been confirmed; C. A friend has been confirmed; D. A colleague has been confirmed; E. A close relative has been suspected; F. A distant relative has been suspected; G. A friend has been suspected; H. A colleague has been suspected; I. None of the above

29. What is the nature of your employment?
A. Employees of state-owned enterprises; B. Employees of private and foreign enterprises; C. Employees of commercial services; D. Employees of transportation; E. Medical and rescue workers; F. Employees of scientific research, education and cultural industries; G. Workers in government agencies; H. Community workers; I. Bosses/entrepreneurs; J. Senior managers of enterprises and institutions; K. Self-employed or freelance; L. Students; M. Farmers; N. Retired; O. Jobless or unemployed; P. Other __________

30. If you are employed, what is your current start-up situation?
A. Working from home; B. The unit has resumed work and I have returned to my place of employment and started work; C. The unit has resumed work and I have returned to my place of employment but I am unable to attend work; D. The unit has resumed work and I am unable to return to my place of employment; E. The unit has not resumed work but will resume work soon; F. The unit has not yet released a date for resumption of work

31. What are your main sources of information on the COVID-19?

	Never	Occasionally	Sometimes	Often	Always
A. Portals (Sina, Baidu, Today’s headlines, etc.)					
B. Central and provincial and municipal governments’ online platforms (websites, microblogs, public numbers)					
C. Central, provincial and municipal media and their online platforms (CCTV, Xinhua News Agency, People's Daily, etc.)					
D. To test whether you have answered carefully, please select “always” for this question only					
E. Other commercial media and magazines in China					
F. Domestic social media (e.g. QQ, Weibo, Douban, etc.)					
G. WeChat groups, friend circles, public websites					
H. Overseas media (including Facebook, Twitter, etc.)					

I.Face-to-face or voice communication with friends and relatives					
J. Push messages from mobile phone text messages					
K. Announcements/notices issued by community radio, bulletin boards, etc.					

32. How do you rate the information on the COVID-19?
A. Very well-informed, with relatively timely access to authoritative and useful information; B. A lot of information, but not much credible and useful information; C. A lot of information, but very confusing in terms of truth and falsity; D. Not enough information

33. With the information available so far, what do you think is the risk of a new lung outbreak?

	Extremely high	High	Moderate	Low	Extremely low
A. Infectious					
B. Lethality					
C. Overall dangerousness					
D. Socially destructive					

34. Some people say that the COVID-19 is a pandemic influenza and that places outside Hubei are overstressed and can be treated at a slightly higher level than ordinary influenza.
A. Don’t agree at all; B. Disagree; C. Agree; D. Agree very much; E. Not sure

35. What are the main preventive measures for you and your family against the epidemic? (Multiple answers allowed)
A. Stay at home and do not go out as much as possible; B. Pay close attention to the information of the epidemic; C. Take your own temperature frequently; D. Wear a mask as much as possible when you go out; E. Clean and disinfect your home frequently; F. Take preventive medicine; G. Exercise more often and always keep an optimistic mood; H. Wash your hands regularly; I. Other

____

36. What is your current physical condition?
A. Extremely healthy; B. Healthy; C. Moderately healthy; D. Unhealthy; E. Extremely unhealthy

37. Are you feeling nervous, anxious and stressed about the COVID-19?
A. Not at all; B. Less often; C. Sometimes; D. More often; E. Very often

38. When do you personally think that the alarm of the COVID-19 will be almost eliminated?
A. In February; B. In March; C. In April; D. In May; E. In June; F. In July; G. Before the end of 2020; H. unknown

39. Are you confident that we will eliminate the COVID-19 outbreak?
A. Extremely confident; B. Confident; C. Unconfident; D. Extremely unconfident; E. Neutral

40. What are the things you or your family would most like to do after the COVID-19 alert is lifted? (Up to three)
A. Eat well; B. Travel well; C. Start a fitness programme; D. Renovate your home to improve hygiene and enjoyment; E. Upgrade your furniture, appliances and internet access; F. Participate in community building to improve the community environment; G. Change your neighbourhood to get a better living environment; H. Own a house in your usual place of residence (only for those who rent a house in their usual place of residence) I. Buy an extra flat to increase risk aversion; J. Be more active in social welfare activities; K. Speak up more to the government; L. Increase psychological construction and psychological counselling; J. Other ___________________

Thank you very much for your support and cooperation!
If you would like to receive a summary report of this research, please leave your email address so that we can give you feedback at
Mail address: ____
Center for Housing and Urban-Rural Development of *Shanghai Jiao Tong University* (https://churd.sjtu.edu.cn)
